# Production and consumption in agri‐food transformations: Rethinking integrative perspectives

**DOI:** 10.1111/soru.12423

**Published:** 2023-01-17

**Authors:** Jonathan D. Beacham, David M. Evans

**Affiliations:** ^1^ University of Bristol Business School Bristol UK

**Keywords:** alternative proteins, agri‐food relations, economy of qualities, edibility, integrative perspectives, transformation, visceral politics

## Abstract

The adverse consequences of contemporary agri‐food relations, particularly in terms of public health and environmental sustainability, have led to growing calls—across interdisciplinary research and policy perspectives—for fundamental systemic change. Focusing on the interconnections and ‘workings’ of agri‐food *systems*, these accounts have coalesced around the vernacular of transformation to think through the possible ways in which these relations might be configured differently. Against this backdrop, the relationship between food ‘production’ and food ‘consumption’ emerges as a key problem. This article revisits debates developed within *Sociologia Ruralis* approximately two decades ago concerning the terms on which consumption and consumers are brought into agri‐food scholarship, arguing that these are given renewed impetus in the context of contemporary calls for agri‐food transformation. We build on and advance these previous integrative efforts both by taking stock of recent advances in consumption studies and by responding to the shifting contours of food politics. The analysis focuses on the case of alternative proteins and outlines three substantive bodies of scholarship—the geographies of edibility, the economy of qualities and visceral politics—that we suggest offer considerable potential for renewing and updating the development of integrative perspectives on production and consumption. To conclude, we reflect on the theoretical and practical risks of seeking to reconcile ‘production’ and ‘consumption’ and argue that these new integrative concepts may themselves provide more suitable conceptual ‘building blocks’ for exploring the transformation of agri‐food relations.

## INTRODUCTION

The adverse consequences of contemporary agri‐food relations—characterised by extensive ecological externalities, bifurcating patterns of malconsumption and malnutrition in majority and minority world contexts, as well as expanding corporate power (Beacham, 2022)—are profound. In recognition of this, growing calls for fundamental change have emerged from both academic (Marsden et al., 2018; Sage, 2014) and policy perspectives (Bhunnoo & Poppy, 2020). Yet whilst change represents a longstanding theme within the broad remit of agri‐food scholarship, expanding streams focus on the prospects for agri‐food *transformation* more specifically. This can be understood as a sustained effort to reconfigure agri‐food *systems* (Béné et al., 2019)—acknowledging the constituent elements and the connections between them—to ‘generate a future where all people have access to healthy diets, which are produced in sustainable and resilient ways that restore nature and deliver just, equitable livelihoods’ (Fanzo et al., 2021, p. 7). Recent contributions (e.g., see Maye & Duncan, 2017) have highlighted divergent understandings of the ways in which these systemic transformations might come about. It remains debated as to whether the ‘top down’ reconfiguration of mainstream, productivist agriculture represents a critical locus, or whether these will be arrived at from the ‘bottom up’ via the transformative potential of alternative food networks, social movements and other geographies of resistance to the status quo (Holt Giménez & Shattuck, 2011). Either way, if questions of systemic transformation encourage timely ‘assess[ments of the] linkages between all food activities, their market and institutional networks; and the nutrition, environment and socio‐economic outcomes’ (Brouwer et al., 2020, p. 1), then the ways in which these interconnections are conceptualised warrant further consideration. This article focuses specifically on the relationship between food ‘production’ and food ‘consumption’, which are domains of activity and analysis that are often treated as separate. We suggest that the development of integrative and holistic perspectives for understanding production and consumption are given renewed impetus in the context of contemporary calls for agri‐food transformations.

In order to advance this argument, we necessarily revisit debates that were initiated within *Sociologia Ruralis* roughly two decades ago. Contributions from *inter alia* David Goodman, Stewart Lockie and their various collaborators (e.g., see Goodman, 2002; Goodman & DuPuis, 2002; Lockie, 2002; Lockie & Kitto, 2000) assessed the ways in which consumption was ‘brought in’ to typically production‐centric agri‐food scholarship in considering the development of more holistic perspectives. Put briefly and bluntly, the emphasis was on developing conceptual frameworks that might allow agri‐food to move beyond economistic and abstract treatments of consumption. Drawing principally on actor‐network theory (ANT) and other relational‐materialist approaches, these integrative efforts rejected the tendencies of traditional political economy approaches that saw power as ‘held’ by specific actors, preferring to theorise these relations as contingent, ‘in process’ and mutually constituted. The point being that explanatory recourse to the dynamics of production alone was insufficient to understand significant trends at the time, notably in the expansion of markets for organic and FairTrade foods as well as more general calls to ‘reconnect’ producers and consumers via short food supply chains (e.g., see; Goodman, 2004; Hudson & Hudson, 2003). In addition to reigniting this agenda in response to the vogue for considering agri‐food transformation, this article also highlights the need to update and develop previous integrative efforts in light of recent advances in consumption studies and the shifting contours of food politics. In doing so, it is not our intention to offer a systemic exploration of production and its relationship with consumption; rather, our aim is to develop ways of thinking across production and consumption. In turn, we ask whether these new ways of thinking might supersede the vernacular of production and consumption in providing more satisfactory framings to conceptualise agri‐food relations^1^ and their possible transformation.

This article proceeds as follows. First, we provide a more detailed discussion of debates surrounding the treatment of consumption and consumers within agri‐food scholarship. Revisiting these debates c.20 years later, we note that the tenor of the original critique rings true today despite the promising contributions developed at the time. With this in place, we turn to consider the changing landscapes of food politics, suggesting that developments in recent years have broadened out questions of where transformations may occur. Accordingly, the analysis that follows focuses on the empirical case of alternative proteins (APs) and their potential role in enabling widespread dietary change. We outline three bodies of scholarship—the geographies of edibility, the economy of qualities and visceral politics—that we suggest offer considerable potential for developing integrative perspectives on production and consumption. To conclude, we consider the conceptual and practical ramifications of bringing these concepts together to theorise the transformation of agri‐food relations, as well as their potential applicability and utility in understanding other empirical developments.

### Integrative understandings: Revisiting the debate, two decades on

The relationship between production and consumption—and the potential that exists to develop integrative perspectives—emerged as a significant area of debate within agri‐food scholarship between the late 1990s and mid‐2000s. This emerged partly in response to the dominant position of political economy perspectives within this scholarship, derived from the empiricist and positivistic legacies of agricultural economics (Smith & Lawrence, 2021). Despite growing recognition that consumption and consumers might indeed be key to understanding worlds of food, political economy accounts gravitated heavily towards explanations grounded in the ‘world’ of production, leading these tending to be theorised in abstract and economistic terms. Consumption was at this point therefore treated as little more than an ‘exogeneous structural category’ (Goodman & DuPuis, 2002, p. 10) generated by—and within—the world of production. Commensurate with this, Lockie suggests that agri‐food scholarship therefore tended to follow two distinct paths, framing consumption ‘either as a set of practices manipulated by capital and the state in the interest of capital accumulation or as the simple agglomeration of individually free and rational choices’ (Lockie, 2002, p. 279). This meant that all too often production was in effect understood either as determining consumption (Evans & Mylan, 2019), or consumption was used in an obverse fashion to discuss production. In short, the integrative agenda sought to interrogate the terms on which understandings of consumption were being ‘brought in’ to agri‐food scholarship.

Wider theoretical currents at the time contributed to the development of the integrative agenda. Against the backdrop of dominant political economy perspectives that favoured structuralist and neoclassical theoretical frameworks, a ‘new’ heterodox sociology of agriculture was taking shape (Smith & Lawrence, 2021). Attempting to move beyond established explanatory frameworks and their blind spots, theorists such as Goodman (2002) drew inspiration from the study of consumption as a social and cultural phenomenon. As Goodman argues, dominant perspectives betrayed widespread advances in social scientific understandings of consumption that had been developed over the preceding decades (cf. Miller, 1995), in turn stymying the development of integrative perspectives. Yet the problem for Goodman, Lockie and their collaborators was not one that could simply be resolved by better acknowledging the existence of ‘the consumer’. Instead, it required the fundamental unpacking of enduring ‘theoretical asymmetries and linearities’ (Goodman, 2002, p. 272) within agri‐food scholarship, seeking to develop new ‘analytical frameworks which [resisted] privileg[ing] the production “moment” in agri‐food circuits’ (Goodman, 2002, p. 272).

In response to this problem, these heterodox perspectives shifted away from conventional political economy approaches to power. Drawing on wider trends in social theory, particularly ANT, a relational‐materialist ontology was advanced. From this perspective, power was no longer something that was simply ‘held’ at certain points in supply chains, contrasted against accounts that defaulted to seeing power as concentrated in the world of production. Instead, power was to be understood in more diffuse terms, contributing to ‘myriad […] strategies, intermediaries or resources, and modes of ordering that are involved in the constitution, construction and maintenance of […] agri‐food networks’ (Lockie & Kitto, 2000, p. 16). This emphasis on networks that were always processually ‘in the making’ allowed for the ‘articulation of concepts that [could] account more adequately for both contingency and regularity along food chains’ (Lockie & Kitto, 2000, p. 4). The integrative agenda therefore rejected dichotomous treatments of ‘production’ and ‘consumption’ as distinct, arguing that ‘greater weight [within analyses] be given to the recursive, relational organization of the socio‐material networks conjoining these “worlds”’ (Goodman, 2002: 273). By this account, who—or indeed what—had agency within agri‐food relations ought not to be assumed or ascribed from the outset. These relational understandings provided an important way in for the integrative agenda to more comprehensively ‘acknowledge consumers as relational actors in recursive, mutually constituted food circuits’ (Goodman, 2002, p. 272) rather than passive actors continually manipulated by the dynamics of production.

Despite the development of promising integrative perspectives for ‘articulating […] production and consumption in a unified framework of analysis’ (Goodman, 2002, p. 276), there have been surprisingly few further contributions since this initial wave of activity (although see, e.g., Evans & Mylan, 2019). Whilst the theoretical sensibilities and methodological habits of relational‐materialist approaches have remained influential in the field of agri‐food scholarship writ large, they have not been fully mobilised to more adequately bring consumption in. Writing approximately two decades on from this initial flurry of activity, we suggest that little has fundamentally changed. A core problem is that consumption remains chronically underdefined. Extrapolating from several developments in consumption scholarship, we suggest that consumption cannot be derived simply and unproblematically from production, nor can it be reduced to a straightforward matter of exchange or consumer choice in the marketplace. We conceptualise consumption here as a process involving the acquisition, appreciation, appropriation, devaluation, divestment and disposal of goods, services and experiences (see Evans, 2019). Crucially, this is a process that occurs within and for the sake of practices (see Warde, 2005) that are configured through the alignment of heterogenous elements including culture, economy, technology and so on. Yet there remains something of a gulf between these advances in understandings of consumption and the ways in which it continues to be framed within the scholarship exploring agri‐food transformation. Despite renewed and persistent calls in interdisciplinary research and policy debates on transformation to better account for consumption (see, e.g., Horton et al., 2017), there has been a reluctance to move beyond the familiar tropes of consumer choice and behaviour.^2^ These tendencies are particularly pronounced within policy agendas, with Brunori et al. (2019) noting the continued reliance on the rationality of consumers to make ‘better’ decisions rather than recasting analyses around structural tendencies. Elsewhere (e.g., see Morgan et al., 2008), transformations in agri‐food relations tend to be attributed to abstract notions of spontaneous and aggregate changes in consumer demand. At the same time, concern with the production ‘moment’ continues to take precedence over consumption, which remains ‘tacked on’ as an afterthought to production‐centric frameworks.

In some respects, the paucity of the ways in which consumption is brought in and treated within agri‐food scholarship is perhaps unsurprising. Consumption studies and agri‐food are, after all, separate fields of enquiry and there is no fundamental reason why the former could be reasonably expected to keep up with developments in the latter or vice versa. By extension, there are clear reasons as to why accounts of food production and food consumption cannot readily be pieced together on symmetrical terms given their divergent theoretical foundations. Yet against this backdrop, it is instructive to note that the evolution of consumption studies over the last c.20 years has brought it to a place where it today shares some common theoretical repertoires with agri‐food scholarship. To elaborate: The relational‐materialist perspectives that Goodman, Lockie and others turned to in the early‐to‐mid 2000s were ahead of theoretical trends within consumption studies. At this point in time, the state of the art in consumption studies was heavily influenced by the so‐called ‘cultural turn’. In the intervening years, there has arguably been a subsequent ‘practice turn’ (see Warde, 2014; cf. Evans, 2020) in consumption studies culminating in the approach and definition that we outline above. Put another way, consumption studies appear to have caught up with the relational‐materialist emphasis of previous integrative efforts within agri‐food scholarship. Given that the operationalisation of more sophisticated accounts of consumption (Evans, 2022) may well dovetail with a substantive interest in systemic transformation within agri‐food scholarship, these domains have much to learn from one another. The growing common ground between them provides further impetus to revisit and update the integrative agenda.

### Changing landscapes of food politics

It is significant that the integrative agenda assumed urgency in the context of a burgeoning interest in ‘alternatives’ in the early‐to‐mid 2000s. Conceptualised as ‘reconnecting’ producers and consumers in novel configurations (Holloway et al., 2007; Marsden et al., 2000; Watts et al., 2005; Winter, 2003; Sonnino & Marsden, 2006), this suite of topics leant themselves readily to questions of integration. The sense of reconnection engendered within an array of different organisational models served to ‘respatialise’ and ‘resocialise’ relations between producers and consumers in ways that were not engendered within mainstream agri‐food relations (Feagan, 2007). Representing a ‘small but important new set of counter‐logics […] stretching out from the main cloth’ (Campbell, 2009, p. 318) and imbued with transformative potential, these alternatives embraced their geographical origins and relations of production at a variety of scales. FairTrade certification, for example, revealed how transnational commodity chains might be ‘done’ differently, empowering rather than disenfranchising distant majority world farmers and producers (Goodman, 2004; Hudson & Hudson, 2003). By placing the lives of these farmers front‐and‐centre in its marketing, the ascendance of FairTrade sought to resocialise relations with Western consumers who were willing to pay price premiums to improve their social and economic conditions. Meanwhile, a tangible concern with the environmental impacts of extensive ‘food miles’ (Lang & Heasman, 2016) at this time saw a strongly renewed emphasis on notions of ‘locality’ within drastically shortened food supply chains (SFSCs), including in farmer's markets, Community Supported Agriculture and direct‐to‐door delivery schemes such as ‘veg boxes’ (Barbera & Dagnes, 2016; Goodman et al., 2012). In contrast with mainstream tendencies of producing food ‘from nowhere’, the interest in SFSCs lay in respatialising relations as food ‘from somewhere’, thereby valorising local and regional dynamics and characteristics (Bové & Dufour, 2001). For Marsden et al. (2000), this loose category of alternatives became entangled in a timely interest in ‘natural’ and ‘better’ food, derived from what was seen as their incipient ‘potential for shifting the production of food commodities out of their “industrial mode” and to develop supply chains that could potentially “short‐circuit” the long, complex and rationally organized industrial chains’ (Marsden et al., 2000, pp. 424–425). In short, the development of these alternatives illuminated the possibility for ofof subverting the status quo and bringing about more progressive states of affairs from the ‘bottom up’.

As Jarosz (2008) notes, these alternatives mobilise a specific ‘politics of consumption’ premised on the ability of consumers to incrementally ‘effect [sic] wide‐ranging transformation of unjust and inequitable social relations of production’ (Jarosz, 2008, p. 234). In turn, the air of passivity hitherto associated with consumption and/or consumers in agri‐food scholarship was simply no longer sufficient to understand the political rationalities of alternatives. The emergence of this politics of consumption around the turn of the millennium further highlighted the problems associated with a dichotomised conceptualisation of production and consumption as discrete ‘worlds’, appearing increasingly untenable and in need of revision, eventually leading to these efforts to map out integrative approaches. Yet despite proliferating interest in alternatives in the years that followed, the integrative agenda did not develop in tandem.

Whilst it is important to understand the context within which the integrative agenda emerged c.20 years ago, it is also worth acknowledging the ways in which this has changed. Alongside now substantive bodies of literature exploring the transformative potential of alternatives in their many guises (e.g., see Maye & Duncan's, 2017, consideration of grassroots social innovations and the transformative capacities of alternative practices), there is growing recognition of a litany of ‘differ[ing] trajectories of agri‐food transition’ (Levidow, 2015, p. 76). Amidst mounting recognition of the extensive externalities of agri‐food (Rust et al., 2020; Stoll‐Kleemann & O'Riordan, 2015; Willett et al., 2019), recent perspectives (e.g., see Herrero et al., 2020) have placed greater emphasis on large scale innovations—technological, organisational and otherwise—to consider their role in enabling widespread dietary change. Recent popular trends (e.g., ‘clean eating’ or the availability of plant‐based ‘mylks’) demonstrate how questions around what and how people eat are increasingly being probed not only by alternatives but also within mainstream settings. Put another way, and the obvious problems with these developments notwithstanding, we suggest that it is the supermarket as much as the farmer's market that represents a likely locus of transformation today. Yet perhaps the most striking example of this food politics is the growth of markets for APs and ‘plant‐based’ alternatives to meat derived from animals. With governments either seeking or being encouraged to enact policies to reduce meat consumption (Dagevos & Voordouw, 2017), APs appear likely to play an important role in enabling these transformations, with the Food and Agriculture Organization recently noting the ‘major opportunities associated with these new food sources […] as we move away from intensive livestock production and overfishing’ (FAO, 2022, para. 3).

The analysis that follows responds to and develops the debates initiated by David Goodman and others c.20 years ago. It considers three discrete—but at times overlapping—bodies of work that we suggest offer significant potential for renewing and updating integrative perspectives on production and consumption. Building on previous integrative efforts (see Goodman, 2002, and articles within), we suggest that paying attention to these bodies of work reveals an array of concepts that more satisfactorily takes stock of recent developments in consumption studies, bringing them ‘in’ to agri‐food scholarship on more equal and symmetrical terms. In order to demonstrate the potential of these bodies of work and their attendant concepts, we focus specifically on the contentious politics of APs and their role in enabling mainstream dietary change. It is to this that we now turn.

### Towards new integrative understandings: The case of APs

Here, we understand APs as foods derived variously from plants, fungi, insects and tissue cultures, which in recent years have been transformed from niche foodstuffs aimed at highly specialised diets to being widely available via mainstream outlets such as supermarkets and fast‐food restaurant chains. Promoted with a range of ‘promissory narratives’ (Sexton et al., 2019) emphasising their transformative capacities, there are growing numbers of companies ‘veganising’ their product ranges (Sexton et al., 2022) as awareness of the various consequences of animal‐derived meat grows (Apostolidis & McLeay, 2016). At the same time, the consumption of animal‐derived meat continues to accelerate in geographically uneven ways, though the gap between minority and majority world contexts is narrowing. Whilst the US has long led the per capita consumption of meat—standing at 121 kg annually in 2013—China has notably seen meat consumption increase by over 18 times in the five decades from 1961 to 2013, latterly at 61 kg per capita (Hansen & Jakobsen, 2020). The rate and scale of global meat consumption carries with it deleterious consequences, from what Weis (2013a) conceptualises as a significant ‘ecological hoofprint’ to a litany of public health implications and beyond. As Weis surmises, there is now an urgent need to confront the ‘social and ecological disaster that is industrial livestock production’ (Weis, 2013b, p. 80).

Against this contradictory backdrop, we note that there are a growing number of countries—including New Zealand, Canada and Switzerland—where per capita meat consumption is now in fact decreasing (Whitton et al., 2021). Such trends have also been recognised within the UK, where the per capita consumption of meat has fallen by 17% over the past decade (Stewart et al., 2021). Meanwhile, ample evidence points to the ascendance of vegetarian, vegan and ‘flexitarian’ diets seeking to reduce or eliminate animal‐derived meat consumption, with the ‘Veganuary’ campaign^3^ attracting record participants year‐on‐year (Sexton et al., 2022). In short, there are nascent signs that we are perhaps reaching ‘peak meat’ (Whitton et al., 2021) as efforts to ‘do’ food differently gain traction.

With global markets for APs growing significantly (Sexton et al., 2019), until relatively recently such shifts may have appeared difficult to imagine. Reports such as the FAO's 2006 *Livestock's long shadow* (Steinfeld et al., 2006) highlight the clear need to reduce meat consumption at a global scale—particularly in minority world contexts—yet the report provides little insight into what might ‘fill the gap’ left by meat in everyday diets. Reflecting on this in 2022, APs have now become so ubiquitous to be noticeable in their absence from these discussions. At the same time, we are inherently cautious of accounts that lurch into treating APs as ‘silver bullet’ solutions to deeply complex problems. For example, APs have environmental impacts associated with their extensive processing (Smetana et al., 2021), and other accounts have noted patterns of co‐option within the portfolios of already powerful agri‐food conglomerates (Sexton et al., 2022). As Sexton et al. elaborate, the marketisation of APs is in many cases unimaginable without Silicon Valley‐led venture capital funding and might therefore be understood as ‘disrupting’ the status quo only in the sense of providing new avenues for capital accumulation for already powerful vested interests. These points are all acknowledged and firmly in hand, we suggest that their contemporary popularisation nonetheless provides an exemplary empirical case to ‘think with’ in developing integrative approaches to food production and food consumption. In order to demonstrate this argument, we first turn our attention to the geographies of edibility scholarship.

#### Geographies of edibility

Understanding something as edible—and thus by extension a food that one could eat—is a fundamental prerequisite in popularising novel and unfamiliar food products. Whilst this may seem like an obvious point, the geographies of edibility scholarship proceeds from the observation that what is in/edible is inherently contingent. In contrast with anthropological perspectives that explore cultural variation in the classification of food and non‐food (Counihan, 1999; Goody, 1982; Mintz & du Bois, 2002), this body of work illuminates the ‘relational process[es] […] positioning […] particular foods as in/edible through mutually implicated practices of production and consumption’ (House, 2018, p. 82). From this perspective, material things do not possess intrinsic characteristics that entail that they are (or are not) a priori edible (Roe, 2006). Conversely, something becoming edible is to be understood as an achievement of a disparate network of connected actors spanning the worlds of production and consumption.

Taking edibility as a crucial interface between the worlds of production and consumption, House's (2018, 2019a, 2019b) exploration of a specific subcategory of APs—insects—is instructive. Whilst regularly eaten in majority world contexts, insects scarcely feature in minority world diets given they do not tend to be understood as an edible food fit for human consumption. Yet there are signs that this situation is changing. Insects as a source of protein have been ‘proposed [as a] solution to the unsustainability of Western meat consumption’ (House, 2018, p. 83) alongside their potential public health benefits. In countries such as The Netherlands, the availability of novel insect‐based foods is testament to the shifting contours of contemporary food politics. The brand Ÿnsect, for example, utilises mealworms and lesser mealworms in the creation of their AdalbaPro Fiber Textured Insect Protein (FTIP). FTIP is then used in place of meat in foodstuffs already familiar to consumers, such as burgers and nuggets. Consequently, these producers primarily target consumers who eat meat but are seeking to reduce their personal consumption through convenient and familiar substitutions. This scholarship demonstrates that these shifts have not occurred spontaneously from the ground up; rather, House (2018) argues that the transformation of insects into something widely understood as edible is an effect of a nascent ‘Dutch edible insect network’. This network includes—but is by no means limited to—academics, policymakers, legislators and regulators, processors, retailers and final consumers themselves.

Within this network, heterogenous elements contribute to making things edible. Some of these elements can be more closely categorised as belonging to the worlds of ‘production’ or ‘consumption’, whilst others span them. For example, technological advancements in food production and processing are self‐evidently important in making insect‐based foods not only edible but actively desirable. But these products are not just technical achievements. Legislation plays an important role in delineating whether insect‐based foods can legally be sold in different contexts, and retailers must stock and promote them as commercially viable products. Experimental consumers also must be encouraged to sample insect‐based foods through re‐working social norms and expectations around eating, as well as ‘buying in’ to discourses and the marketing of these products. Zirp Insects, an Austrian insect‐based snack food manufacturer provides a key example of how products are positioned as both ‘good for you’ and ‘good for the planet’: ‘compared to conventional animal foods, insects are an ecologically sensible source of protein and protect our environmental resources’ (author's translation). Hence, what is at stake here is not merely a malleable array of consumer preferences, but a mechanism ‘by which consumers are responsibilised to become “good” eaters’ (Sexton, 2018, p. 1). Indeed, making things edible does not occur through a ‘straightforward psychological repositioning [by consumers] of particular foods within an otherwise unchanged social landscape’ (House, 2019a, p. 1294), but it is a distributed process potentially engendering new practices taking hold in an ethical register.

In sum, the geographies of edibility scholarship are useful as an integrative perspective in moving beyond dichotomised treatments of ‘production’ and ‘consumption’. Whilst we note that edibility—understood here as the ontological status of ‘things’ becoming ‘food’ (Roe, 2006)—is a comparatively low threshold to be met, it would be wrong to suggest that the availability of insect‐based foods has necessarily led to sustained changes in consumption patterns in countries such as The Netherlands, where these food products remain relatively niche (House, 2019b). Put another way, edibility does not entail popularity. Nonetheless, recasting analyses around relationally constituted networks shows how upstream ‘fixes’ in the world of production cannot hope to resolve problematic tendencies in the world of consumption if foods do not reach a minimum threshold of being seen as edible. Though we note important critiques of these network‐based approaches (see, notably, Fine, 2004) and their inherent risk of failing to provide sufficient clarity of causal relations between constituent elements, this discussion reveals how in/edibility can be mobilised to shift the locus of responsibility for sustainable transformations towards other actors—principally ‘the consumer’ (Barnett et al., 2011; Evans et al., 2017). By the same token, it also shows how consumers are actively implicated in the shifting of these relations and ought not to be treated as passive dupes or mere handmaidens to powerful corporate or legislative actors.

#### The economy of qualities

In order to make sense of widespread and potentially more sustained changes, it is important to understand not just how things become (novel) foods (House, 2019a) but also the terms on which they are valued or otherwise determined to be good (cf. Heuts & Mol, 2013). The economy of qualities perspective developed by Callon et al. (2002) is particularly instructive in this regard. Its starting point is the idea that qualities are never simply observed. Rather, they result from qualification processes, which aim to ‘establish a constellation of characteristics, stabilized at least for a while, which are attached to the product and transform it temporarily into a tradable good in the market’ (Callon et al., 2002, p. 199). Viewed as such, there is an important and helpful distinction to be drawn between *intrinsic* qualities of food (material and physical attributes that can be measured) and the *extrinsic* evaluation (subjective judgement) of these (Murdoch & Miele, 2004). In their analysis of the market for orange juice, for example, Evans and Mylan (2019) suggest that ‘freshness’ is at once a key coordinating principle and an inherently unstable category. Accordingly, they point to qualification as a process that involves a focus on the characteristics that must be considered for orange juice to be considered fresh (intrinsic) but also the terms on which ‘freshness’ is positioned favourably within the economy of qualities (extrinsic).

Expanding markets for APs demonstrate the importance of the economy of qualities. There has been a recent proliferation of food producers—including De Vegetarische Slager (The Vegetarian Butcher^4^) in The Netherlands, Impossible Foods and Beyond Meat in the USA (California, specifically), and THIS in the UK—that purport to mimic or replicate, rather than simply approximate, meat. Within their marketing, these companies are unified by their explicit ambitions for their products to be categorised and qualified *as* ‘meat’. For example, the Vegetarian Butcher^5^ states that they offer ‘high quality meat’ that is ‘made by meat lovers, for meat lovers’ with the ambition of ‘replicating that moreish taste and texture you get from meat’. In order to do this, a wide range of strategies have been adopted. These include the development of specific products that correspond to specific meats (‘pork’, ‘chicken’ and ‘beef’ rather than just, e.g., ‘Quorn’) and making a concerted effort to ensure that these alternatives are positioned alongside their conventional counterparts (on ‘the fresh meat aisle’^6^) in retail spaces.^7^ Most importantly, they have focused on what makes meat taste *like meat* 8 with a view to replicating this using alternative protein sources, simultaneously accounting for other material attributes like fat content, ‘skins’ for sausages, texture, how it behaves when cooked (browning, suitability for barbecuing), and even burgers that ‘bleed’. These products are qualitatively different from other, earlier, efforts to develop meat substitutes. In the case of burgers, for example, they stand in stark contrast to those that bear no resemblance to meat (mashed‐up pulses and vegetables in breadcrumbs) and are far more faithful to ‘real’ meat than burgers shaped from a generic textured vegetable protein. The core marketing proposition,^9^ then, is for products that are ‘meat’—retaining all the extant or purported benefits—but *better* in terms of health, environmental sustainability and animal welfare.

Turning to consumption, it is fair to suggest that the literature on qualification (Callon et al., 2002; cf. contributions to Beckert & Aspers, 2011; Beckert & Musselin, 2013) has focused principally on how ‘supply side’ actors position goods and propositions in markets. Despite clear acknowledgement that consumers are central to success in the economy of qualities, they are invoked principally to talk about ‘producers’ (echoing Goodman's critique of agri‐food scholarship, see Evans & Mylan, 2019). Nevertheless, by emphasising that qualification involves a distributed apparatus of cognition, Callon et al. (2002) leave space open for further developments that place consumption more squarely at the centre of analysis. Notable examples include Cochoy's (2008) work on the quality‐based ‘calculations’ that consumers make; Smith Maguire's (2013) work on the qualification of wine, which demonstrates how particular qualities are negotiated and accomplished; and Evans & Mylan's (2019) demonstration of how qualification cuts across sites and spaces of production and consumption. Accepting that consumers are economic actors who are actively involved in qualification processes, it follows that this is a useful concept for thinking across ‘production’ and ‘consumption’ without privileging one domain of practice at the expense of the other.

It stands to reason that the successful qualification of APs not only as meat, but as *good* meat, rests on the extent to which consumers individually and collectively categorise, value and attach themselves to these propositions in the market (cf. Cochoy et al., 2017). Studies of how consumers categorise meat substitutes emphasise that new products need to resemble meat if they are to be successful in replacing it (Elzerman et al., 2011; Hoek et al., 2011). Given the strategic focus and attendant technical advances that accompany the aforementioned AP ‘successors’^10^ to meat, it seems credible that they will be categorised as meat by consumers, both in terms of their physical attributes and their ability to replace ‘conventional’ meat within existing culinary repertoires. In terms of what makes these products better than conventional meat, there are well‐established links (e.g. Apostolidis & McLeay, 2016) between the development of the market for APs and growing consumer concerns surrounding the ethical and environmental consequences of terrestrial livestock production. Given the shifting contours of contemporary food politics, it seems likely that the cultural frames are already in existence to help position plant‐based favourably within the economy of qualities. The expanding markets for APs evince the idea that their qualification as ‘good meat’ is completed by and through practices of consumption. Finally, we note that the success of these products cannot be attributed to their price. On the contrary, in a typical UK supermarket, the price of a pack of ‘Beyond Meat’ burgers is approximately three times as much as the ‘standard’ (middle) product line. Setting aside concerns about access and affordability—or the rather more pertinent question of why meat derived from animals is comparatively cheap (Carolan, 2018)—this further underscores the point that values for money might be superseding value for money in the economy of qualities (cf. Lang, 2010).

Again, it would be naïve to read the individual and collective responses of consumers to these marketing propositions as a simple matter of manipulation and coercion by the food industry. Even setting aside the powerful counternarratives from the incumbent meat and dairy industries, we note that doing so would repeat the mistake of deriving consumption from production and eliding decades of considered scholarship into the process and content of consumption. As a minimum, we note wider cultural changes—principally related to concerns around the consequences of eating animal‐derived meat (Sanchez‐Sabate & Sabaté, 2019)—that not only transform the practices for which (food) consumption occurs but also the frameworks of symbolic value that support the qualification of APs as ‘good’ meat in people's everyday lives beyond the moment of market exchange.^11^

#### Visceral politics

Even when foods are understood as edible and ‘good’, an important question remains: How do foods become normalised and widely accepted over time? Placing embodiment centrally in considering this problematic, the visceral politics scholarship argues that food–body relationships themselves are historically contingent. Developed principally through the work of Allison and Jessica Hayes‐Conroy (Hayes‐Conroy & Hayes‐Conroy, 2010a; Hayes‐Conroy & Hayes‐Conroy, 2010b; Hayes‐Conroy & Martin, 2010; Hayes‐Conroy & Hayes‐Conroy, 2013; Hayes‐Conroy, 2014), this work argues that political economies mediate embodied responses to different foods. This means that there is nothing inherently ‘natural [or] pre‐political [about any of these] bodily impulses’ (Hayes‐Conroy, 2014, p. 188), but food–body relations can be attuned to political‐economic configurations in different ways over time. Indeed, what ‘good’ food is, or might be, is never entirely settled. Encouraging a ‘concurrent awareness of the structural, epistemological, and material forces’ that taken across different scales ‘affect food judgements and behaviours’ (Hayes‐Conroy & Hayes‐Conroy, 2013, p. 88), the visceral politics scholarship highlights the centrality of the body in understanding the relationship between production and consumption, as well as how these relations change.

Acknowledging the centrality of the body is significant for any attempt to bring ‘consumption’ in to agri‐food scholarship given that food, after all, is not something people simply contemplate. As Carolan argues, ‘we think, and thus socially construct, *with our bodies*’ (Carolan, 2008, p. 408, emphasis ours). In contrast with the ideational tendencies of Western thought, experiences of food are ultimately *biosocial*. They are as much about the embodied responses to different flavours, textures, sensations and so on as it is the constituent micro‐ and macronutrients that any given food provides. Despite the intimate nature of these experiences, this does not entail that they ought to be considered solely in individual terms nor the reality of biological or physiological responses to food be denied. Conversely, the visceral politics scholarship argues that food–body relationships result from ‘a complex interplay between the body, the material stuff of food, and the social […] meaning we give food’ (Watson & Cooper, 2021. p. 112). As such, whilst bodies become attuned to specific political economies as ‘normal’, these relationships are never deterministic. A degree of contingency entails that there is always the possibility of innovations spurring the development of new and experimental food–body relationships.

A historical example of the ways in which food–body relationships can change is useful to illustrate this point at a conceptual level. Canning technologies were originally developed with military logistics in mind but were extended to civilian markets in the early 20th century. For reasons of practicality, they readily found traction amongst consumers in countries such as the US and England (Carolan, 2011). However, canned food initially received a distinctly lacklustre reception in French society. Despite legitimate health concerns arising from early attempts at canning (e.g., cases of botulism and lead poisoning), ‘improvements to the canning processes did nothing to make people like or even trust canned foods because trust first requires experience to build upon’ (Carolan, 2011, p. 34). Put bluntly, Bruegel (2002) notes how the French found canned food ‘repugnant’, and it was rejected almost *en masse*. For Carolan, ‘the French, in a word, *had to be tuned to the tastes* and practical requirements of canned foods […] [and] *bodies had to be conditioned to “choose” these foods*’ (Carolan, 2011, pp. 33–34, emphasis ours). In other words, embodied judgements matter, and physiological responses cannot be understood in isolation from social or cognitive dynamics. Attuning French bodies to the pleasures of industrialised food production epitomised by canning technologies took significantly longer than their American or English counterparts, for whom such tastes did not attract such strong reactions. Today, canning technologies are commonplace around the world and normalised as a method of food preservation.

This example conceptually illustrates the links between embodied visceral judgements and wider political economies, which are intricately connected as responses are shaped by the social—or more specifically political and economic—context in which they are formed. Relating this back to APs specifically, Sexton (2016) details her decision to purchase, cook and subsequently eat grilled ‘Beyond Chicken’ strips, constituted predominately of soy protein but seeking to emulate chunks of chicken meat. Sexton suggests that it was only through cooking with the strips that she could see them ‘perform as meat’, unable to ‘accept the strips as “meat” until the point of eating them’ (Sexton, 2016, p. 74). The centrality of these ‘visceral and embodied encounters’ (Sexton, 2016, p. 72) is instructive for understanding contemporary shifts in relations of food production and consumption. As we have noted, the rate and scale of animal‐derived meat consumption in many minority world countries is historically anomalous, yet is still understood by many as normal or even ‘natural’. From a visceral politics perspective, it would be wrong to assume that people enjoy meat due to their wilful ignorance of the consequences or that their preferences are in some capacity ‘false’ or misguided. The question is not why actors find animal‐derived meat ‘tasty’ but the conditions under which these subjective preferences come to be formed. As Sexton's encounter with the Beyond Chicken strips reaffirms, shifting these preferences implicates a wide range of biosocial mechanisms embedded within, and derived from, historical power relations. Whilst these never wholly determine consumption practices, this discussion highlights the ways in which visceral politics are subject to contestation by producers and consumers alike.

This discussion highlights a further contentious dimension of APs. As previously mentioned, recent years have seen considerable effort dedicated to the development of APs that accurately replicate the visceral experience of eating animal‐derived meat, arguably best exemplified by burgers made to ‘bleed’ via the inclusion of soy leghemoglobin or beetroot juice. Yet from some perspectives, questions remain as to whether APs should at an ethical level be attempting to reinforce the biosocial mechanisms that normalise meat eating as pleasurable and desirable, that is, in eating juicy and bloody flesh. As Siegrist and Hartmann (2020) note, APs are still associated with feelings of ‘unnaturalness’ and repulsion to a significant proportion of ‘carnist’ meat eaters, even though a growing proportion of the same group evoke feelings of disgust when shown images of meat (Becker & Lawrence, 2021). Although we can only speculate as to how these visceral politics will change in the future, this discussion highlights the centrality of food–body relationships in understanding changing relations of production and consumption, as well as what is at stake in determining what foods are understood to be ‘normal’, ‘good’ and actively desirable.

## DISCUSSION AND CONCLUSION

This article has revisited debates around integrative perspectives on food production and food consumption. Contemporary interest in systemic transformation within agri‐food scholarship provides a clear impetus for this task. On the one hand, these systemic conceptualisations of agri‐food represent a promising development in encouraging the recognition of interconnections between constituent elements rather than defaulting to accounts that privilege the production ‘moment’. Though sustained engagement with the dynamics of ‘production’ clearly remains crucially important, the terms on which consumption has been brought into these debates remain limited and attest to the enduring strength of ‘theoretical asymmetries and linearities’ (Goodman, 2002, p. 272) within agri‐food scholarship and associated policy perspectives. This article has therefore revisited these debates precisely on the grounds that the tenor of Goodman's original critique surrounding the denigrated position of consumption to a large degree still holds true. In tracing the contours of contemporary food politics through a case study of APs, we have reviewed recent theoretical advances in consumption studies to reignite this agenda and establish some foundations for further contributions. As we have demonstrated, the geographies of edibility, the economy of qualities and visceral politics all present their own distinct ways of thinking across ‘production’ and ‘consumption’ rather than reifying them as self‐evident analytical categories.

At this juncture, we note that there are risks—both political and conceptual—of seeking to reconcile ‘production’ and ‘consumption’ within a systemic framing. Most presciently, the enduring lack of analytical precision around what consumption ultimately *is*, as well as on what terms it is understood as discrete from production (Evans, 2020; Graeber, 2011), can lead to problematic tendencies. For example, dominant policy agendas have tended to equate consumption with the rhetorical figure of ‘the consumer’ (see Evans et al., 2017) who might be encouraged to make ‘better’ choices at the point of purchase. Whilst slippages between ‘consumption’ and ‘consumers’ may on the face of it appear innocuous or a matter of semantics, this elision risks further responsibilising individual consumers *vis‐à‐vis* processes of systemic transformation. Given these concerns, as well as an open question about the extent to which it is plausible—or even desirable—to integrate the two ‘wholes’ of production and consumption (see Evans, 2019), we suggest there is a need to look beyond the integrative ‘project of articulating food production and consumption in a unified framework of analysis’ (Goodman, 2002, p. 276). Despite the convenience of the ‘production–consumption’ vernacular, the political and conceptual problems associated with its usage cause us to ask whether it represents the most useful conceptual starting point for exploring the transformation of agri‐food relations. We propose that it may make more sense to position the geographies of edibility, the economy of qualities and visceral politics *not* as integrative concepts that simply more satisfactorily span and account for the dynamics of these ‘worlds’ but ultimately offer potential for pushing past and superseding these vernacular framings. We do not suggest that these concepts are exhaustive nor do we claim a comprehensive treatment of how they might span the worlds of ‘production’ and ‘consumption’. Rather, our intention has been to highlight the renewed importance of this debate and to invite further theoretical and empirical contributions that continue in this vein.

The integrative perspectives that we outline here nevertheless offer unique insights into how foods come to be valued and normalised in different contexts, and in different ways, over time. In this regard, they offer significant potential in being applied beyond our empirical focus here on APs specifically. For example, we acknowledge growing interest in a range of related empirical examples including new markets for ‘waste’ food products, the development of new bioprocesses such as precision fermentation, automated additive processing (colloquially known as ‘3D printed’ food), regenerative agriculture and land use change to name but a few of a potentially much longer list of examples. Of course, the scope and focus of the integrative perspectives that we explore here entail that they ‘speak’ to these empirical phenomena in their own distinct ways and to varying extents. Whilst a fuller discussion of this is beyond the scope of this article, we note, for example, the ways in which edibility is a core problem for the marketisation of 3D printed food, such as in the Israeli company Plantish's development of 3D printed ‘salmon’ fillet, including fish ‘skin’. A different example highlights the ways in which foods become seen as ‘good’—per the economy of qualities—is key in foods that problematise notions of ‘waste’, such as in the creation of ‘superflours’ from spent brewery grain by Rise Products in the US. Further, even dynamics such as changing patterns of agricultural land use are implicated here. For example, visceral attunement to ‘meaty’ diets is premised on significant amounts of land being dedicated to terrestrial livestock and cereals to feed these animals and is, as we have already noted, historically anomalous. Driving this point home, food manufacturers such as Hodmedod, based in the East of England, are promoting and growing plant‐based protein (beans, such as fava, and various pulses) that are congruent with production practices more characteristic of the Iron Age, when these were regularly grown in this geographical region. This raises intriguing questions over what can be understood as from a visceral politics perspective as a ‘normal’ diet, the ways in which these expectations change over time, as well as the iterative relationship between the dynamics of ‘production’ and ‘consumption’. Taken together, these integrative concepts tesselate in ways that highlight the importance of exploring bodies, markets and materials in tandem (cf. Evans et al., 2022). To this end, we speculate that attention to questions such as these within a renewed integrative agenda might unlock new ways of thinking about the transformation of agri‐food relations in profoundly different configurations.

## CONFLICT OF INTEREST

The authors have no conflicts of interest to declare.

## Data Availability

Data sharing is not applicable to this article as no datasets were generated or analysed during the current study.

## ENDNOTES

1 Throughout the article, we mobilise ‘agri‐food relations’ over alternative framings in focusing our analysis on the ‘relationships between practices associated with the provision of food and the consumption of that food’ (Lockie & Kitto, 2000, p. 3). As a point that we return to, divisions of academic labour that have separated the study of agricultural production from the study of food consumption are increasingly untenable, with ‘agri‐food’ representing an important recognition of their interconnection. Furthermore, we maintain a critical distance from the vernacular ‘systemic’ framing given the risks of this framing in downplaying social, cultural and political dimensions of how these relations change (see Sanderson Bellamy & Ioris, 2017).

2 Typifying the limited framings through which consumption tends to be theorised within this scholarship, Horton et al. (2017) argue that ‘[c]onsumers [provide] feedback through their influence on stakeholder behaviour and the external socio‐political factors’ (p. 198). Surprisingly, this point does not receive further elaboration or explanation.

3 The aim of Veganuary is to follow a fully vegan diet for the month of January. In the UK, it is now widely promoted within supermarkets, celebrity‐endorsed cookbooks, alongside other media promotions, with over 620,000 participants taking part in 2022. See: https://veganuary.com/.

4 A highly successful Dutch company that was acquired by Unilever in 2018.

5 See https://www.thevegetarianbutcher.co.uk.

6 For example, see https://edition.cnn.com/2019/06/09/business/beyond‐meat‐seth‐goldman/index.html.

7 This has become legally contentious in recent years, with powerful members of the food industry lobbying to reserve names such as ‘burgers’ and ‘sausages’ exclusively for products containing meat. See, for example: https://www.foodmanufacture.co.uk/Article/2020/10/26/MEPs‐reject‐ban‐on‐plant‐free‐sausage‐and‐burger‐tags.

8 See https://impossiblefoods.com.

9 Which is of course to say nothing about the veracity of these claims. As noted, the environmental and public health benefits of APs are deeply contested.

10 See https://www.thevegetarianbutcher.co.uk/our‐story.html.

11 See above our definition of consumption that emphasises *inter alia* ‘appropriation’ and ‘appreciation’ as well as ‘acquisition’.
